# Health biotechnology development in Malaysia: targeting local and global health needs through health technology

**DOI:** 10.1186/1471-2458-14-S1-O23

**Published:** 2014-01-29

**Authors:** Gulifeiya Abuduxike

**Affiliations:** 1 United Nations University International Institute for Global Health (UNU-IIGH), 56000 Cheras, Kuala Lumpur, Malaysia

## Background

In Malaysia, biotechnology is identified as one of enablers to accelerate the transformation of the country into knowledge based economy and industrialised country by 2020. Since 2005, the government has established national strategic plans, funding mechanisms and implementation agencies to develop this sector. Aim of this research was to examine the development of this industry and to evaluate the capabilities and resources that can be leveraged to address the local and global health needs while ensuring the economic benefit to the country.

## Materials and methods

Health innovation ecosystem framework was applied and case study approach was used for this research. Quantitative and qualitative information were gathered through intensive literature reviews, face to face interviews, expert group discussions and survey questionnaires. Respondents were selected by convenient sampling method from the public and private health biotechnology related sectors in Malaysia.

## Results

The findings from this research were summarised into four major points which were: 1) success stories and factors influencing the success in Malaysia context; 2) main features and niche areas of the sector; 3) main challenges and weaknesses of Malaysian health biotechnology sector 4) identification of opportunities and strategic recommendations for the sustainable development of health biotechnology sector in Malaysia.

## Conclusions

Biotechnology sector is at its infant stage of development due to the remaining challenges and weaknesses in the health innovation system. Thus, enormous work and efforts are needed from various sectors in all aspects to achieve the nation’s 2020 targets. The main priority should be given to the development of the basic or fundamental research areas by adjusting the education system and to provide the necessary skill sets for the sustainable development of this knowledge driven industry.

